# Terpenoids from the Octocorals *Menella* sp. (Plexauridae) and *Lobophytum crassum* (Alcyonacea)

**DOI:** 10.3390/md10020427

**Published:** 2012-02-15

**Authors:** Cheng-Hung Lee, Chia-Ying Kao, Shih-Yao Kao, Chih-Han Chang, Jui-Hsin Su, Tsong-Long Hwang, Yueh-Hsiung Kuo, Zhi-Hong Wen, Ping-Jyun Sung

**Affiliations:** 1 Department of Biomedical Engineering, National Cheng Kung University, Tainan 701, Taiwan; Email: 298f@vghtc.gov.tw (C.-H.L.); cchang@mail.bme.ncku.edu.tw (C.-H.C.); 2 Department of Nutrition, HungKuang University, Taichung 433, Taiwan; 3 Department of Orthopedics, Taichung Veterans General Hospital, Taichung 407, Taiwan; 4 Graduate Institute of Marine Biotechnology and Department of Life Science and Institute of Biotechnology, National Dong Hwa University, Pingtung 944, Taiwan; Email: chiaying1229@gmail.com (C.-Y.K.); sweetcloud0906@gmail.com (S.-Y.K.); x2219@nmmba.gov.tw (J.-H.S.); 5 National Museum of Marine Biology and Aquarium, Pingtung 944, Taiwan; 6 Department of Marine Biotechnology and Resources and Division of Marine Biotechnology, Asia-Pacific Ocean Research Center, National Sun Yat-sen University, Kaohsiung 804, Taiwan; 7 Graduate Institute of Natural Products, Chang Gung University, Taoyuan 333, Taiwan; Email: htl@mail.cgu.edu.tw; 8 School of Chinese Pharmaceutical Sciences and Chinese Medicine Resources and Tsuzuki Institute for Traditional Medicine, College of Pharmacy, China Medical University, Taichung 404, Taiwan; Email: kuoyh@mail.cmu.edu.tw

**Keywords:** menelloide, germacrane, *Menella*, lobocrassin, cembrane, *Lobophytum*, elastase

## Abstract

A new germacrane-type sesquiterpenoid, menelloide E (**1**), and a new cembrane-type diterpenoid, lobocrassin F (**2**), were isolated from the octocorals *Menella* sp. and *Lobophytum crassum*, respectively. The structures of terpenoids **1** and **2** were determined by spectroscopic and chemical methods and compound **2** was found to display a significant inhibitory effect on the release of elastase by human neutrophils.

## 1. Introduction

Octocorals have been proven to be rich sources of natural terpenoid derivatives [1,2] and terpenoid analogues are often found in large amounts in marine invertebrates, and represent the largest percentage of natural products isolated from marine organisms [3]. In a continuation of our search for new substances from marine invertebrates collected off the waters of Taiwan at the intersection of the Kuroshio current and the South China Sea surface current, the chemical constituents of a specimen of gorgonian identified as *Menella* sp. (Plexauridae) [4,5] were studied. Its organic extract displayed inhibitory effects on the generation of superoxide anion (inhibition rate 84.7%) and the release of elastase (inhibition rate 96.2%) at a concentration of 10 µg/mL. We further isolated a new germacrane-type sesquiterpenoid, menelloide E (**1**), from *Menella* sp. Furthermore, a new pyrancembranoid diterpenoid, lobocrassin F (**2**), was isolated from the octocoral *Lobophytum crassum* (Alcyonacea). In this paper, we describe the isolation, structure determination and bioactivity of terpenoids **1** and **2**. 

## 2. Results and Discussion

### 2.1. Isolation and Structure Determination of Menelloide E *(**1**)* from *Menella* sp.

**Figure 1:**
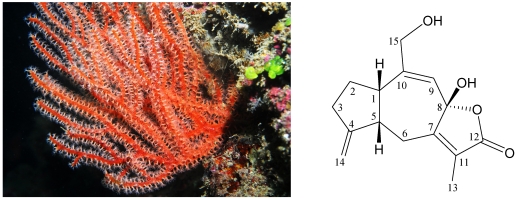
The octocoral *Menella* sp. and the structure of menelloide E (**1**).

Previous studies on the chemical constituents of octocorals belonging to genus *Menella* (Figure 1) have afforded a series of interesting secondary metabolites [6,7,8,9,10,11,12,13,14,15,16,17], including sesquiterpenoids, menelloides A–D [14,15]. Menelloide E (**1**) was isolated as a colorless oil (Figure 1), and the molecular formula for this compound was determined to be C_15_H_18_O_4_ (seven units of unsaturation) using HRESIMS (C_15_H_18_O_4_ + Na, *m/z* 285.1105, calculated 285.1103). Comparison of the ^13^C NMR and DEPT data with the molecular formula indicated that there were two exchangeable protons, which required the presence of two hydroxy groups. This deduction was supported by a broad absorption in the IR spectrum at 3413 cm^−1^. The IR spectrum also showed a strong band at 1746 cm^−1^, consistent with the presence of an ester group. The ^13^C NMR data for **1** confirmed the presence of 15 carbon signals (Table 1), characterized by DEPT as a methyl, four sp^3^ methylenes, an sp^2^ methylene, two sp^3^ methines, an sp^2^ methine, an sp^3^ quaternary carbon and five sp^2^ quaternary carbons. A suite of resonances at δ_C_ 175.1 (C-12), 149.8 (C-7), 127.4 (C-11), 102.7 (C-8) and 8.7 (CH_3_-13), could be assigned to the α-methyl-α,β-unsaturated-γ-lactone moiety. Two additional unsaturated functionalities were indicated by ^13^C resonances at δ_C_ 154.5 (C-10), 111.4 (CH-9), 157.6 (C-4) and 104.5 (CH_2_-14), suggesting the presence of a trisubstituted olefin and an exocyclic carbon-carbon double bond. Thus, from the reported data, the proposed skeleton of **1** was suggested to be a sesquiterpenoid with three rings. 

**Table 1:** NMR spectroscopic data (500 MHz, CDCl_3_) for menelloide E (**1**).

Position	δ_H_ (*J* in Hz)	δ_C_, Mult.
1	2.85 td (11.5, 8.0)	48.3, CH
2α/β	1.19 m; 1.74 m	28.7, CH_2_
3	2.25 m	33.8, CH_2_
4		157.6, qC
5	3.21 m	40.7, CH
6α/β	3.00 dd (15.0, 3.5); 3.51 dd (15.0, 3.5)	26.2, CH_2_
7		149.8, qC
8		102.7, qC
9	5.68 s	111.4, CH
10		154.5, qC
11		127.4, qC
12		175.1, qC
13	1.93 s	8.7, CH_3_
14a/b	4.89 s; 4.66 s	104.5, CH_2_
15a/b	3.67 dd (10.5, 5.5); 3.61 dd (10.5, 5.5)	70.2, CH_2_
OH-8	2.49 s	
OH-15	2.04 t (5.5)	

**Figure 2:**
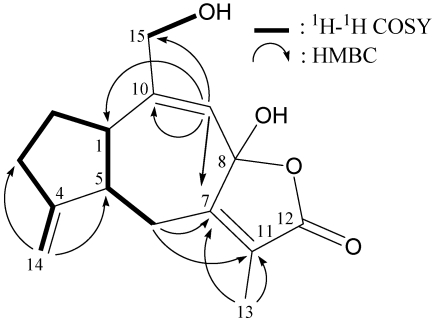
The ^1^H–^1^H COSY and selective key HMBC correlations (^1^H→^13^C) for sesquiterpenoid **1**.

From the ^1^H–^1^H COSY spectrum of **1** (Figure 2), it was possible to differentiate between the separate spin systems of H-1/H_2_-2/H_2_-3, H_2_-3/H_2_-14 (by allylic coupling), H-1/H-5/H_2_-6 and OH-15/H_2_-15. These data, together with the HMBC correlations between H-14a/C-3, C-5; H-14b/C-3; H-6β/C-7, C-11; H-9/C-1, C-7, C-10, C-15; and H_3_-13/C-7, C-11 (Figure 2), permitted elucidation of the carbon skeleton. The exocyclic carbon-carbon double bond at C-4 was confirmed by the HMBC correlations between H-14a/C-3, C-5 and H-14b/C-3; and further supported by the allylic couplings between H_2_-3 and H_2_-14. The methylene unit at δ_C_ 70.2 was correlated to the methylene protons at δ_H_ 3.67 and 3.61 in the HMQC spectrum and the H-9 olefinic proton signal was ^3^*J*-correlated with C-15 (δ_C_ 70.2), proving the attachment of a hydroxymethyl group at C-10. Thus, the remaining hydroxy group should be positioned at C-8 and concluded to be a part of a hemiketal constellation on the basis of a characteristic carbon signal at δ_C_ 102.7 (C).

**Figure 3:**
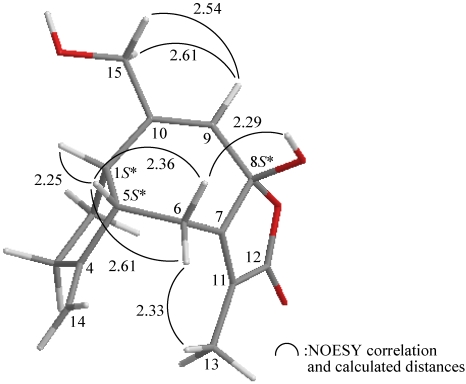
The computer-generated model of **1** using MM2 force field calculations and the calculated distances (Å) between selected protons with key NOESY correlations.

The relative configuration of **1** was elucidated mainly from a NOESY spectrum as being compatible with that of **1** offered by computer modeling (Figure 3), in which the close contacts of atoms in space calculated were consistent with the NOESY correlations [18]. In the NOESY experiment of **1**, H-5 exhibited a correlation with H-1, indicating that these two protons (H-1 and H-5) were situated on the same face and assigned as β protons. One proton of C-6 methylene (δ_H_ 3.51) exhibited correlations with H-1 and OH-8 (δ_H_ 2.49), but not with H_3_-13, indicating that this proton and the hydroxy group at C-8 were β-oriented in **1** by molecular modeling analysis (Figure 3). This observation was supported by a correlation between H-6α (δ_H_ 3.00) and H_3_-13, but not with OH-8, although a correlation was also found between H-5 and H-6α. The *Z*-configuration of the C-7/11 double bond was elucidated from a correlation between H-6α and the C-13 vinyl methyl. Correlations observed between H-9 and H-15a/b reflected the *E* geometry of the double bond at C-9/10. From the above evidence, the relative configurations of the chiral carbons of **1** were assumed to be 1S*, 5S* and 8S*. On the basis of the above analysis, the relative structure of **1** was determined. 

### 2.2. Isolation and Structure Determination of Lobocrassin F *(**2**)* from *Lobophytum crassum*

**Figure 4:**
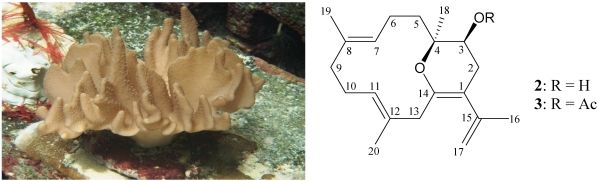
The octocoral *Lobophytum crassum* and the structures of lobocrassin F (**2**) and its derivative 3-*O*-acetyllobocrassin F (**3**).

The octocoral *L. crassum* (Figure 4), distributed in the waters of Taiwan has afforded various new cembranoids [19,20,21,22,23,24,25,26], including lobocrassins A–E [24]; new glycolipids [27]; and new α-tocopherols [28]. The molecular formula for lobocrassin F (**2**) (Figure 4) was determined to be C_20_H_30_O_2_ (six units of unsaturation) using HRESIMS (C_20_H_30_O_2_ + Na, *m/z* 325.2145, calculated 325.2143). Comparison of the ^13^C NMR and DEPT data with the molecular formula indicated that there was an exchangeable proton, which required the presence of a hydroxy group. This deduction was supported by a broad absorption in the IR spectrum at 3265 cm^−1^. The ^13^C NMR data for **1** confirmed the presence of twenty carbon signals (Table 2), characterized by DEPT as four methyls, six sp^3^ methylenes, an sp^2^ methylene, an sp^3^ methine, two sp^2^ methines, an sp^3^ quaternary carbons and five sp^2^ quaternary carbons. Compound **2** was determined to possess a tetrasubstituted olefin (δ_C_ 108.2, C-1; 144.2, C-14), two trisubstituted olefins (δ_C_ 131.5, C-12; 130.0, C-8; 127.8, CH-7; 127.7, CH-11) and an exocyclic carbon-carbon double bond (δ_C_ 144.3, C-15; 113.7, CH_2_-17). The above functionalities accounted for four of the seven degrees of unsaturation, suggesting a bicyclic structure of **2**.

**Table 2:** NMR spectroscopic data (500 MHz, CDCl_3_) for lobocrassin F (**2**).

Position	δ_H_ (*J* in Hz)	δ_C_, Mult.
1		108.2, qC
2α/β	2.01 dd (16.5, 6.5); 2.39 dd (16.5, 4.0)	31.5, CH_2_
3	3.54 br s	67.7, CH
4		77.6, qC
5	1.60 m	35.0, CH_2_
6	2.21 m	23.4, CH_2_
7	5.11 t (7.5)	127.8, CH
8		130.0, qC
9	2.05 m	39.5, CH_2_
10	2.15 m	25.6, CH_2_
11	5.02 t (7.5)	127.7, CH
12		131.5, qC
13α/β	2.98 d (14.0); 2.72 d (14.0)	41.1, CH_2_
14		144.2, qC
15		144.3, qC
16	1.81 s	22.7, CH_3_
17a/b	4.72 d (1.5); 4.93 d (1.5)	113.7, CH_2_
18	1.17 s	16.5, CH_3_
19	1.59 s	15.6, CH_3_
20	1.43 s	15.7, CH_3_

**Figure 5:**
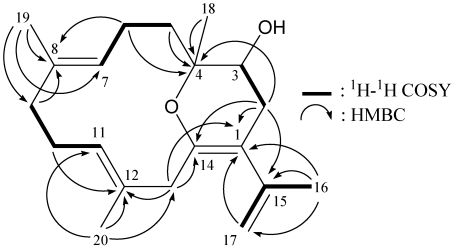
The ^1^H–^1^H COSY and selective key HMBC correlations (^1^H→^13^C) for diterpenoid **2**.

From the ^1^H–^1^H COSY spectrum of **2** (Figure 5), it was possible to identify the separate spin systems of H_2_-2/H-3, H_2_-5/H_2_-6/H-7, H_2_-9/H_2_-10/H-11 and H_3_-16/H_2_-17 (by allylic coupling). These data, together with the HMBC correlations between protons and quaternary carbons of **1**, such as H_2_-2, H_2_-13, H_3_-16, H_2_-17/C-1; H_2_-2, H_2_-5, H_2_-6, H_3_-18/C-4; H_2_-6, H_2_-9, H_2_-10, H_3_-19/C-8; H_2_-10, H_2_-13, H_3_-20/C-12; H_2_-2, H_2_-13/C-14; and H_2_-2, H_3_-16/C-15 (Figure 5), permitted elucidation of the carbon skeleton. The vinyl methyls attached at C-8, C-12 and C-15 were confirmed by the HMBC correlations between H_3_-19/C-7, C-8, C-9; H_3_-20/C-11, C-12, C-13; and H_3_-16/C-1, C-15, C-17 (Figure 5). However, because no HMBC correlation was observed between H-3 and C-14, the remaining hydroxy group was positioned at C-3, and an ether bridge was determined to be located between C-4 and C-14 to form a pyran ring. In order to confirm this ratiocination, acetylation of **2** was performed and its derivative 3-*O*-acetyllobocrassin F (**3**) (Figure 4) was obtained in high yield (95%). This result indicates that a secondary hydroxy group attached at C-3 in **2** is necessary. 

**Figure 6:**
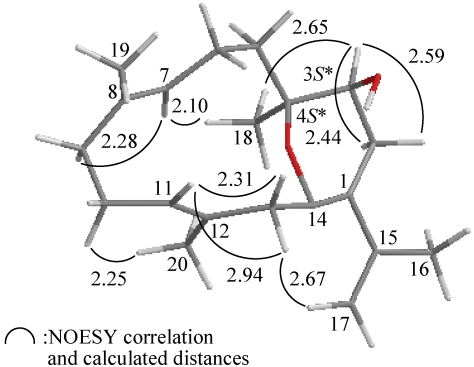
The computer-generated model of **2** using MM2 force field calculations and the calculated distances (Å) between selected protons with key NOESY correlations.

The relative configuration of **2** was elucidated mainly from a NOESY spectrum as being compatible with that of **2** offered by computer modeling (Figure 6), in which the close contacts of atoms in space calculated were consistent with the NOESY correlations [18]. In the NOESY experiment for **2**, correlations were observed between H-3 and H_3_-18, suggesting that these protons are located on the same face and can be assigned as α protons. Correlations observed between H-7/H_2_-9 and H-11/H_2_-13, as well as the lack of correlation between H-7/H_3_-19 and H-11/H_3_-20, reflected the *E* geometry of the double bonds at C-7/8 and C-11/12. Additionally, H-17a (δ_H_ 4.72) was correlated with H-13α (δ_H_ 2.98), suggesting an *s-cis* diene moiety in **2**. On the basis of the above findings, the structure of **2** was elucidated and the chiral carbons of **2** were assigned as 3S* and 4S*.

In previous studies, cembranoids possessing an ether linkage to form a pyran or a tetrahydropyran rings have rarely found [29,30,31,32]. To the best of our knowledge, lobocrassin F (**2**) is the first pyrancembranoid possessing a C-4/14 ether linkage to be obtained from soft corals belonging to the genus *Lobophytum*.

The *in vitro* anti-inflammatory effects of terpenoids **1** and **2** were tested (Table 3). Diterpenoid **2** (lobocrassin F) was found to show a significant inhibitory effect on the release of elastase by human neutrophils. 

**Table 3:** Inhibitory effects of terpenoids **1** and **2** on the generation of superoxide anion and the release of elastase by human neutrophils in response to FMLP/CB.

	Superoxide anion			Elastase release	
Compounds	IC_50_ (µg/mL)	Inh % *^a^*		IC_50_ (µg/mL)	Inh % *^a^*
**1**	>10	19.85 ± 6.65		>10	26.99 ± 4.99
**2**	>10	7.80 ± 5.23		6.27 ± 1.91	58.29 ± 5.47
DPI *^b^*	0.82 ± 0.31				
Elastatinal *^b^*				31.82 ± 5.92	

*^a^* Percentage of inhibition (Inh %) at a concentration of 10 µg/mL; *^b^* DPI (diphenylene indoniumn) and elastatinal were used as reference compounds.

## 3. Experimental Section

### 3.1. General Experimental Procedures

Optical rotations were measured on a Jasco P-1010 digital polarimeter. Infrared spectra were recorded on a Varian Diglab FTS 1000 FT-IR spectrometer; peaks are reported in cm^−1^. NMR spectra were recorded on Varian Inova 500 or Varian Mercury Plus 400 NMR spectrometers using the residual CHCl_3_ signal (δ_H_ 7.26 ppm) as the internal standard for ^1^H NMR and CDCl_3_ (δ_C_ 77.1 ppm) for ^13^C NMR. Coupling constants (*J*) are given in Hz. ESIMS and HRESIMS were recorded using a Bruker APEX II mass spectrometer. Column chromatography was performed on silica gel (230–400 mesh, Merck, Darmstadt, Germany). TLC was carried out on precoated Kieselgel 60 F_254_ (0.25 mm, Merck); spots were visualized by spraying with 10% H_2_SO_4_ solution followed by heating. HPLC was performed using a system comprised of a Hitachi L-7100 pump, a Hitahci L-7455 photodiode array detector and a Rheodyne injection port. A normal phase column (Hibar 250 × 10 mm, Merck, silica gel 60, 5 µm) was used for HPLC.

### 3.2. Animal Material

#### 3.2.1. *Menella* sp.

Specimens of the gorgonian coral *Menella* sp. were collected by trawling off the coast of southern Taiwan at a depth of 100 m in December 2004 and stored in a freezer until extraction. A voucher specimen (NMMBA-TW-GC-005) was deposited in the National Museum of Marine Biology and Aquarium, Taiwan. This organism was identified by comparison with previous descriptions [4,5]. 

#### 3.2.2. *Lobophytum crassum*

Specimens of the soft coral *L. crassum* were collected by hand using scuba equipment off the coast of northeast Taiwan at a depth of 10 m in August 2007 and stored in a freezer until extraction. A voucher specimen (NMMBA-TW-SC-2007-33) was deposited in the National Museum of Marine Biology and Aquarium, Taiwan. This organism was identified by comparison with previous descriptions [4,5]. 

### 3.3. Extraction and Isolation

#### 3.3.1. *Menella* sp.

The gorgonian coral *Menella* sp. (wet weight 451 g) was collected and freeze-dried. The material was minced and extracted with ethyl acetate (EtOAc) at room temperature. The EtOAc layer was separated on silica gel and eluted using *n*-hexane/EtOAc (stepwise from 100:1 to 0:100 *n*-hexane/EtOAc) to obtain fractions 1–16. Fraction 13 was separated by normal-phase HPLC (NP-HPLC), using mixtures of *n*-hexane and EtOAc (1:2–pure EtOAc) to yield fractions 13A–13Q. Fraction 13I was further purified by NP-HPLC using a Hibar silica gel 60 column (250 × 10 mm, Merck, 5 µm) developed with a mixture of *n*-hexane and EtOAc (1:1, flow rate: 2.0 mL/min) to yield menelloide E (**1**, 1.0 mg, *t*_R_ = 51 m).

Menelloide E (**1**): colorless oil; [α]^25^_D_ +9 (*c* 0.05, CHCl_3_); IR (neat) ν_max_ 3413, 1746 cm^−1^; ^1^H (CDCl_3_, 500 MHz) and ^13^C (CDCl_3_, 125 MHz) NMR data, see Table 1; ESIMS: *m/z* 285 (M + Na)^+^; HRESIMS: *m/z* 285.1105 (calcd for C_15_H_18_O_4_ + Na, 285.1103).

#### 3.3.2. *Lobophytum crassum*

The soft coral *L. crassum* (wet weight 1.3 kg) was collected and freeze-dried. The material was minced and extracted with EtOAc at room temperature. The EtOAc layer was separated on silica gel and eluted using *n*-hexane/EtOAc (stepwise from 100:1 to 0:100 *n*-hexane/EtOAc) to obtain fractions 1–12. Fraction 4 was separated by NP-HPLC, using mixtures of *n*-hexane and EtOAc to yield fractions 4A–4G. Fraction 4C was re-purified by NP-HPLC using a Hibar silica gel 60 column (250 × 10 mm, Merck, 5 µm) developed with a mixture of *n*-hexane and EtOAc (22:1, flow rate: 2.0 mL/min) to yield lobocrassin F (**2**, 2.8 mg, *t*_R_ = 134 m).

Lobocrassin F (**2**): colorless oil; [α]^25^_D_ +20 (*c* 0.1, CHCl_3_); IR (neat) ν_max_ 3265 cm^−1^; ^1^H (CDCl_3_, 500 MHz) and ^13^C (CDCl_3_, 125 MHz) NMR data, see Table 2; ESIMS: *m/z* 325 (M + Na)^+^; HRESIMS: *m/z* 325.2145 (calcd for C_20_H_30_O_2_ + Na, 325.2143).

Acetylation of Lobocrassin F (**2**): Lobocrassin F (**2**) (0.5 mg) was stirred with 1 mL of acetic anhydride in 1 mL of pyridine for 4 h at room temperature. After evaporation of excess reagent, the residue was separated by column chromatography on Si gel to give 3-*O*-acetyllobocrassin F (**3**) (0.54 mg, 95%); ^1^H (CDCl_3_, 400 MHz) δ_H_ 5.05 (1H, t, *J* = 7.2 Hz, H-7), 4.99 (1H, t, *J* = 7.2 Hz, H-11), 4.91 (1H, br s, H-17b), 4.77 (1H, dd, *J* = 8.0, 6.0 Hz, H-3), 4.71 (1H, br s, H-17a), 2.88 (1H, d, *J* = 10.0 Hz, H-13α), 2.79 (1H, d, *J* = 10.0 Hz, H-13β), 2.45 (1H, dd, *J* = 16.8, 5.6 Hz, H-2β), 2.19 (2H, m, H_2_-6), 2.09 (2H, m, H_2_-10), 2.06 (3H, s, acetate methyl), 2.04 (2H, m, H_2_-9), 1.99 (1H, dd, *J* = 16.8, 8.4 Hz, H-2α), 1.78 (3H, s, H_3_-16), 1.61 (2H, m, H_2_-5), 1.57 (3H, s, H_3_-19), 1.42 (3H, s, H_3_-20), 1.11 (3H, s, H_3_-18).

### 3.4. Molecular Mechanics Calculations

Implementation of the MM2 force field [18] in CHEM3D PRO software from CambridgeSoft Corporation (Cambridge, MA, USA; ver. 9.0, 2005) was used to calculate molecular models.

### 3.5. Superoxide Anion Generation and Elastase Release by Human Neutrophils

Human neutrophils were obtained by means of dextran sedimentation and Ficoll centrifugation. Measurements of superoxide anion generation and elastase release were carried out according to previously described procedures [33,34]. Briefly, superoxide anion production was assayed by monitoring the superoxide dismutase-inhibitable reduction of ferricytochrome *c*. Elastase release experiments were performed using MeO-Suc-Ala-Ala-Pro-Valp-nitroanilide as the elastase substrate.

## 4. Conclusions

In our previous studies, a series of sesquiterpenoids was isolated from the gorgonian *Menella* sp. Of these compounds, germacrane-type sesquiterpenoids with a hydroxymethyl group, such as compound **1** (menelloide E), are rarely found in marine organisms. As described in the Introduction, the organic extract of *Menella* sp. displayed significant inhibitory effects on the generation of superoxide anions and the release of elastase. However, at this stage, the results showed that the isolated compounds only exhibited weak activity or were not active in anti-inflammatory activity testing [13,14,15]. We suggest that active components exist in other fractions, and these fractions will be studied in the future. The interaction among these isolates will also be studied if we are able to obtain sufficient amounts of the metabolites. It is worth noting that the cembranoids possessing a C-4/14 ether linkage to form a pyrancembranoid, such as **2** (lobocrassin F) were discovered for the first time. All the corals are claimed to be threatened species, and we therefore want to obtain and culture these interesting specimens as sources of potential natural products. However, owing to their structural complexity, it is difficult to obtain sufficient amounts of the bioactive metabolites, such as lobocrassin F (**2**) and lobocrassin B [24], for further study of their potential medicinal usage. The octocorals *Menella* sp. and *L. crassum* have begun to be transplanted in tanks using our highly-developed aquaculture technology for the extraction of natural products in order to establish a stable supply of bioactive material, including extracts and pure compounds. A study focusing on the chemical constituents of a cultured soft coral, *Lobophytum crassum*, has been performed, and three new tetrahydrofuran cembranoids, culobophylins A–C, were isolated from this cultured marine organism [25]. 
